# Efficient CRISPR/Cas9-mediated Targeted Mutagenesis in Populus in the First Generation

**DOI:** 10.1038/srep12217

**Published:** 2015-07-20

**Authors:** Di Fan, Tingting Liu, Chaofeng Li, Bo Jiao, Shuang Li, Yishu Hou, Keming Luo

**Affiliations:** 1 Key Laboratory of Eco-environments of Three Gorges Reservoir Region, Ministry of Education, Chongqing Key Laboratory of Transgenic Plant and Safety Control, Institute of Resources Botany, School of Life Sciences, Southwest University, Chongqing 400715, China; 2 Key Laboratory of Adaptation and Evolution of Plateau Biota, Northwest Institute of Plateau Biology, Chinese Academy of Sciences, 810008 Xining, China

## Abstract

Recently, RNA-guided genome editing using the type II clustered regularly interspaced short palindromic repeats (CRISPR)-associated protein (Cas) system has been applied to edit the plant genome in several herbaceous plant species. However, it remains unknown whether this system can be used for genome editing in woody plants. In this study, we describe the genome editing and targeted gene mutation in a woody species, *Populus tomentosa* Carr. *via* the CRISPR/Cas9 system. Four guide RNAs (gRNAs) were designed to target with distinct poplar genomic sites of the phytoene desaturase gene 8 (*PtoPDS*) which are followed by the protospacer-adjacent motif (PAM). After *Agrobacterium*-mediated transformation, obvious albino phenotype was observed in transgenic poplar plants. By analyzing the RNA-guided genome-editing events, 30 out of 59 PCR clones were homozygous mutants, 2 out of 59 were heterozygous mutants and the mutation efficiency at these target sites was estimated to be 51.7%. Our data demonstrate that the Cas9/sgRNA system can be exploited to precisely edit genomic sequence and effectively create knockout mutations in woody plants.

The CRISPR/Cas9 system is highly efficient at generating targeted mutations in stable transgenic poplar plants, and homozygous mutations at the desired sites can be created in the first generation.In recent years, genome editing technologies using engineered nucleases have been developed as effective genetic engineering methods to target and digest DNA at specific locations in the genome. To date, there are three main types of engineered nucleases for genome editing: zinc finger nuclease (ZFN), transcription activator-like effector nuclease (TALEN), and CRISPR (clustered regularly interspaced short palindromic repeats)/Cas^1^. Among these genome editing approaches, due to its simplicity, design flexibility, and high efficiency, the CRISPR/Cas9 system has now been utilized widely for editing the genome of various organisms, including bacteria, yeast and animals^2^. Most recently, it has also been reported to successfully and specifically edit the genome in plants^3^,^4^.

Since the first reports of CRISPR/Cas9 directed genome editing in two model plant species, *Arabidopsis thaliana*^5^,^6^ and *Nicotiana benthamiana*^6^,^7^, this system has been shown to effectively work in at least five crop species, including monocots: rice^5^,^8^, wheat^9^,^10^, sorghum^11^, maize^12^ and dicots: tomato^13^. The transient expression of Cas9 and sgRNA successfully directs genome modifications in leaf cells or protoplast of *Arabidopsis*^5^ and tobacco^7^,^11^, and in protoplast of wheat^10^. The stable transformation process is also applied to create genetically 1 edited lines carrying a mutation in the gene of interest in all these plant species using CRISPR/Cas9^4^. Moreover, in the stable transgenic lines of *Arabidopsis*, rice and tomato, the sequence changes in first generation of transformants were persist in the next generation^14^,^15^. Although CRISPR/Cas9 is rapidly becoming a primary choice for gene editing in plants, there is no report on the efficacy of CRISPR/Cas9 in woody species yet. Therefore, more extensive investigation is still needed to determine whether the efficacy of this system will be universal.

As a most widely planted fast-growing tree, poplars have tremendous economic and ecological value. Since the whole-genome sequence of *Populus trichocarpa* was released in 2006^16^, extensive genomic resources are now available for functional genomics studies in this species, which has been used as a model in forest genetics and woody plant studies. Thus, understanding of the molecular mechanisms of gene function and transcriptional regulation in *Populus* is crucial for genetic engineering in trees and sustainable forest management. Compared to *Arabidopsis*, rice and other annual model plants, however, functional genomics researches in woody plants are more difficult due to long vegetative periods, low efficiency of genetic transformation and a limited number of mutants^17^. Despite some promising approaches to create knockout mutants in poplar^18^, large-scale gene mutant resources are still lacking so far.

Here we report an improved approach to delivery Cas9 coding gene and multiple sgRNAs into plant cells by a single plasmid. Based on this system, an poplar endogenous phytoene desaturase gene (*PtoPDS*) was disrupted site-specifically in the first generation of transgenic plants. The mutagenesis mediated by Cas9/sgRNA in poplar was highly efficient and both homoallelic and heteroallelic *pds* mutant were detected by DNA sequencing. Taken together, our data suggest that the CRISPR/Cas9 system is a highly efficient and powerful tool for genome modifications in woody plants.

## Materials and Methods

### Growth and transformation of *P. tomentosa* Carr. plants

*Populus tomentosa* Carr. (clone 741) is grown in the greenhouse at 25 °C under a 14-/10-h light/dark cycle with supplemental light (4500 lx). Leaf discs from *P. tomentosa* Carr. were transformed as described previously by Jia *et al*.^19^. Leaves of Chinese white poplar (*P. tomentosa* Carr.) were excised from *in vitro* plantlets, cut into disks and dipped in the diluted *Agrobacterium* culture for 8–10 min. After excess liquid on the surface was absorbed by sterilized paper, the leaf disks were transferred to woody plant medium (WPM) (2 mg/L zeatin, 1 mg/L 1-naphthalene acetic acid [NAA]). The infected disks were co-cultivated in dark for 2 days and then transferred to callus-inducing medium containing 2 mg/L zeatin, 1 mg/L NAA, 400 mg/L cefotaxime, 9 mg/L hygromycin and 0.8% (w/v) agar. After 2–3 weeks of culture without light, these leaf disks with induced calli were subcultured on screening medium (2.0 mg/L zeatin, 0.1 mg/L NAA , 400 mg/L cefotaxime, 9 mg/L hygromycin and 0.8% [w/v] agar) to induce adventitious buds. Regenerated shoots were transferred to rooting medium, containing 0.1 mg/L NAA, 400 mg/L cefotaxime and 9 mg/L hygromycin. Transgenic plants were selected with 9 mg/L hygromycin.

### Cloning of *PtoPDS* gene

The genomic DNA fragment of *PtoPDS* was amplified with gene-specific primers (*PtoPDS*-F: 5´-GTTGAATTTGGTTTTGGAGAAATG-3´; *PtoPDS-R*: 5´- CATTTAATGGTGCAGGGAGAAC-3´) designed based on its homologous gene (Potri.014G148700) sequence in the *P. trichocarpa* by PCR. The PCR reaction was carried out with pfu DNA polymerase (Takara, China) in a total volume of 50 μL at 94 °C for 3 min; 32 cycles of 94 °C for 45 s, 56 °C for 45 s and 72 °C for 90 s, followed by a final extension of 72 °C for 10 min. The PCR product was cloned and sequenced. The sequence result was supplemented in online materials.

### CRISPR/Cas9 target sites selecting

The sequence of *PtoPDS* was input in the online tool ZiFiT Targeter Version 4.2 (http://zifit.partners.org/ZiFiT/Introduction.aspx)^20^, which could find the CRISPR/Cas9 target sites within an input sequence. Four of output target sites were selected for designing the sgRNA sequences based on their location in gene and their GC content.

### Assemble Cas9/sgRNA construct

The binary pYLCRIPSR/*Cas9* multiplex genome targeting vector system carrying CAS9 coding gene and four plasmids with sgRNA cassettes driven by *AtU3b*, *AtU3d*, *AtU6-1* and *AtU6-29*, respectively, and the multiple sgRNA assembly instruction based on Golden Gate Cloning^21^,^22^, were provided by Yao-Guang Liu of South China Agricultural University^23^. A CRIPSR/*Cas9* construct carrying four sgRNA cassettes was generated.

### Genomic DNA extraction

For analysis the mutation of PDS gene in transgenic T0 poplars, the genomic DNA was extracted from stable transgenic and wild-type plants following a typical CTAB method. About 0.1 g tissues of poplars were ground in liquid nitrogen and 400 μL of pre-heated CTAB buffer was added for each sample. After incubated at 65 °C for 30 min, 200 μL of chloroform was added and the resulting mixtures stay in room temperature for 10 min. After centrifugation at 16,000 *g* for 5 min, the supernatant was transferred to a new tube, mixed with 300 μL of isopropanol and incubation in 4 °C for 30 min. Then, genomic DNA was precipitated by 16,000 *g* centrifuge for 10 min and the DNA pellet was washed with 0.5 mL of 70% ethanol. Pellet of genomic DNA was dissolved in 100 μL of H_2_O and concentration was determined using spectrophotometer.

### Detection of mutation

The genomic DNA extracted from poplars was then used as template to amplify the endogenous PDS fragment by PCR. PCR was performed using specific primers for each targets: the pair of *PtoPDS*-F1: 5´- GTTGAATTTGGTTTTGGAGAAATG -3´ and *PtoPDS-R1*: 5´- GCGGAGAAGAACGAAAGG -3´ cover the region of target site 1, 2 and 3; the pair of *PtoPDS*-F2: 5´- TAGAGGCAGTGAATCAATGGG -3´ and *PtoPDS*-R2:5´- CCTAAAACATCTCTTGCTTCAAGC -3´ cover the region of target site 2, 3 and 4. The PCR product was separated on an ethidium bromide-stained agarose gel (1.5%) and bands were recovered and cloned into the pMD19-T Simple vector (Takara, China). The mutantions were identified through Sanger sequencing of individual clones. All sequence results were compared with the reference sequence of *PtoPDS* gene by alignment in DNAMAN (version 7.0). The mutation rate in transgenic plants was calculated according to the ratio of mutated clonal amplicons versus total sequenced clonal amplicons.

## Results

### Strategy for detection the CRISPR/Cas9-mediated mutagenesis of poplar endogenous gene

**Figure 1:**
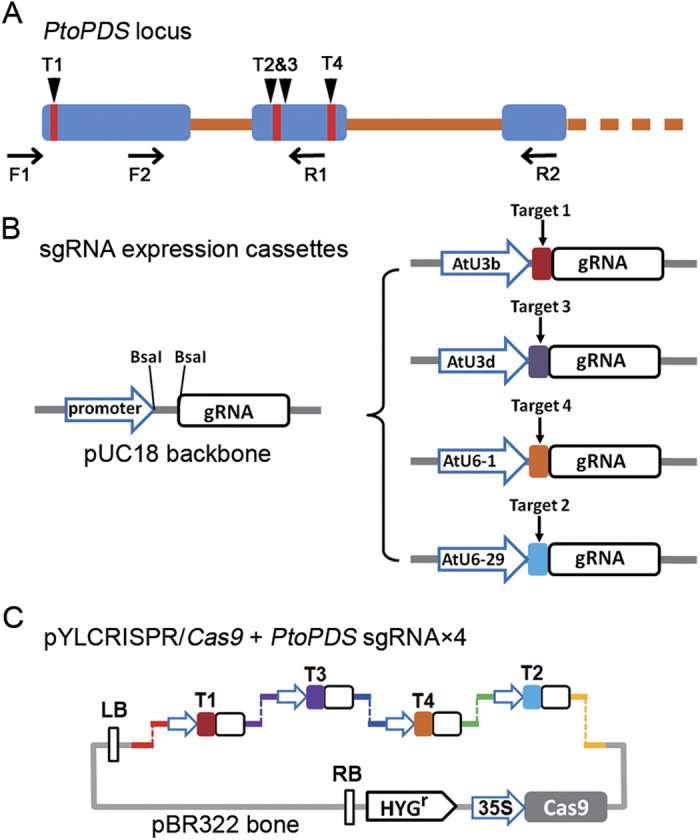
Schematic diagram of assembling Cas9/sgRNA construct and selecting target sites in the *PtoPDS* gene. (**A**) Schematic illustrating the four sgRNAs (red lines) targeting the *PtoPDS* coding sequence. Blue boxes indicate exons; orange lines indicate introns; target sites 2 and 3 are titllingly arranged. F1, F2, R1, R2 indicate binding sites of the primers using for PCR amplification. (**B**) Schematic view of the method for constructing the expression cassettes of sgRNAs. Left, the backbone of sgRNA that any specific targeting sequence can be inserted between the promoter and the unchanged part of guide-RNA using *Bsa*I. Right, the four promters from *Arabidopsis*, *AtU3b*, *AtU3d*, *AtU6-1*, *AtU6-29* were used to drive the four *PtoPDS* targeted sgRNAs, respectively. (**C**) Schematic diagram of the assembling of sgRNAs and Cas9 expression cassettes in a single binary vector for plant stable transformation mediated by *Agrobacterium*. By the design tails after cutting with *Bsa*I, four sgRNA expression cassettes were ligated into the binary vector sequentially.

In order to test whether the CRSPR/Cas9 system could effectively direct gene-specific editing in *Populus*, we selected the poplar phytoene desaturase gene (*PtoPDS*) , which is required for chlorophyll biosynthesis and its mutant shows an albino phenotype in other plant species^9^,^24^, as the target of Cas9 endonuclease. Four 20-bp sequences with tandem guanosine nucleotides (PAM) on their 3´-regions in *PtoPDS* locus were elected as sgRNA complementary sites, including one in 5´ of first exon and three in the second exon (Supplementary Figure S1 and Fig. 1A). By a two-step assemble strategy, the four targeting sequences aiming to *PtoPDS* were first inserted in the sgRNA expression cassettes (Fig. 1B), and then their cassettes were combined with the Cas9 endonuclease coding sequence in a single plant binary vector, pYLCRIPSR/Cas9P35S-H (Fig. 1C). Using this system and *Agrobacterium*-mediated transformation, Cas9 and four sgRNAs could simultaneously express in transgenic poplar.

### CRIPSR/Cas9-mediated mutagenesis of *PtoPDS* in transgenic poplar

**Figure 2:**
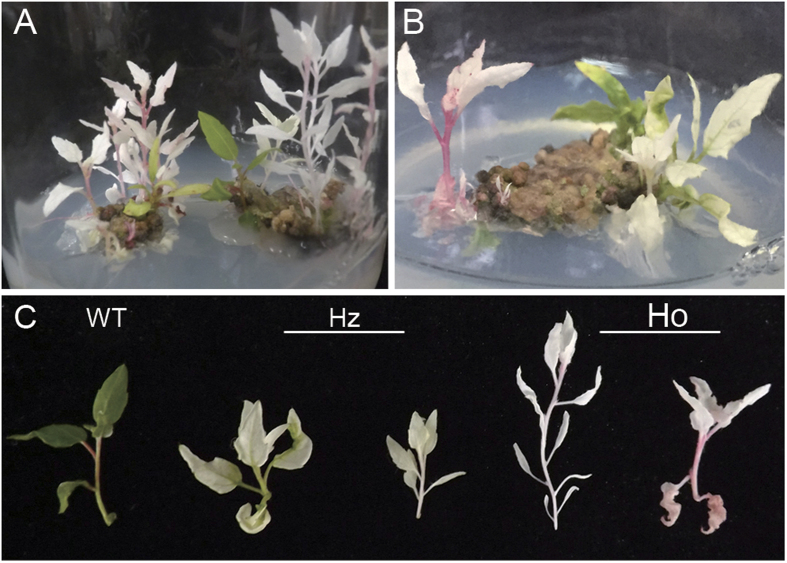
Albinism phenotype of transgenic poplars. (**A,B**) Albino phenotype of the regenerated poplar shoots generated on the CRISPR/Cas9-*PtoPDS* transformed leaf discs. (**C**) Representative CRISPR/Cas9 directed *PtoPDS* plants (T0). WT, Nontransgenic wild-type poplar; Ho, biallelic homozygous mutant; Hz, biallelic heterozygous mutant. Both Ho and Hz plants show an albino phenotype.

We found that, most (89%) of leaf discs transformed with the pYLCRIPSR/*Cas9*+sgRNA generated at least one albino shoots, which lost green color in the whole plants (Fig. 2). Because the *PDS* gene is a necessary member in chlorophyll biosynthesis, albino phenotype in these transgenic plants indicates the loss of *PtoPDS* function. The ratio of albino phenotype occurred in T0 transgenic poplar is more than 50% (30 out of 59), much higher than that previous reported in other species such as *Arabidopsis*, rice, wheat, sorghum and tomato^4^,^6^,^8^,^9^,^13^,^14^, while no albino phenotype was observed in the control transformed with an empty vector (Table 1).

### The poplar *PDS* gene was mutated at desired targets

**Figure 3:**
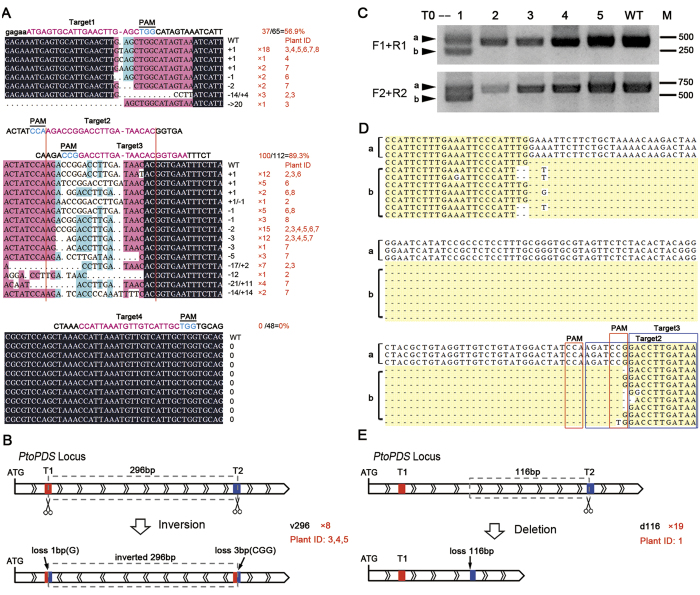
CRISPR/Cas9-mediated gene editing in transgenic poplar plants. (**A**) A range of insertion and deletions (indels) were found in the other T0 plants. Red number in right side means the no. of detected clones with such mutant allele and in which plant such mutant allele is found. (**B**) A big fragment inversion mutant of *PtoPDS*. The sequence between the target site1 and target site 2 (296 bp) of *PtoPDS* were cut down by Cas9 and ligated back to the genome reversely, with 1 and 3 bp of nucleotides around the up- and downstream cut sites were lost. (**C**) DNA samples from independent transgenic poplar were analyzed for mutations by PCR assays. In the top row, F1 and R1 were used; in the bottom row, F2 and R2 were used. T0-1 represents a heterozygous mutant of *PDS*, in which two amplification bands with different length were obtained in this assay: “**a**” indicates the full-length fragment of the *PDS* gene, “**b**” indicates a shorter fragment of the *PDS* gene with mutation. (**D**) The PCR products from T0-1 plant were separated by their size and cloned and sequenced to validate the deletion directed by CRISPR/Cas9 in the PDS gene. (**E**) A big fragment deletion mutant of *PtoPDS*. The 116 bp sequence upstream of target site 2 was lost in the DNA repairing after Cas9 mediated double strain break. In **B** and **E**, Red and blue quadrangles indicate the target site 1 and 2 in *PtoPDS* locus respectively; the red number in the right side means the no. of detected clones with such mutant allele.

To further verify whether loss of green in the transgenic poplar was caused by generation of mutations in the *PtoPDS* gene by the CRISPR/Cas9 system, more than 100 clones from 8 independent transgenic T0 plants were randomly selected for sequencing (Table S1). The results confirmed that all of these transgenic plants with albino phenotype contained mutant alleles in the *PDS* gene. Most of the *PDS* mutant alleles were small insertions or deletions (indels, less than 15 bp) 1 at the desired target sites, as the consequence of repairing through non-homologous end joining (NHEJ) following sgRNA-directed Cas9 cleavages (Fig. 3A). However, some alleles with a sequence inversion (Fig. 3B) between two sgRNAs targeting sites or a big fragment deletion (Fig. 3C–E) were also detected in our experiments. As a result of sequence rearrangement in the exons, the translation of the endogenous *PDS* gene was frame-shifted or prematurely terminated in the Cas9+*PtoPDS* sgRNAs transgenic T0 poplars. Interestingly, we also found that the contributions of the four selected sgRNAs to directing Cas9 and mutating target gene were not equal, and the highest efficiency (89.3%) of mutagenesis appeared in the sites T2&T3 of the target gene, middle in the site T1 (56.9%) and no mutation was detected in the site T4 (Fig. 3A and Table S1), indicating that the appropriate selection of sgRNA pairs is important to effectively generate indels.

In addition, at least two copies of the *PDS* gene in Chinese white poplar genome were amplified by the gene-specific primers we used, and they could be distinguished based on the nucleotide polymorphism in their sequences flanking the target regions (Figure S2). The Cas9 could direct mutation on both of the two *PtoPDS* copies in a single transgenic plant (Figure S2). This suggests the ability of this multiple sgRNA-Cas9 system to knock-out two or multiple loci in the poplar genome at once.

### The Cas9/sgRNA system generated both biallelic homozygous and heterozygous mutants in transgenic poplar

Sequencing analysis of randomly selected clones from individual transgenic T0 plants revealed that, among the 65 clones that could cover target site 1, 2 and 3, most (61) of them were mutated in at least one of these three target sites (Fig. 3A). Actually, 7 (Plant ID 2–8) out of 8 transgenic T0 plants analyzed, contained only mutated alleles of the *PtoPDS* gene, suggesting that these transgenic lines should be biallelic homozygous *pds* mutants (Table S1). In contrast, for another transgenic T0 plant (Plant ID 1) which exhibited the half-white phenotype in their 1 organs (Fig. 2B,C), two bands were observed (Fig. 3C) when amplified with the gene-specific primers for the poplar *PDS* gene. Sequencing analysis confirmed the presence of the innate allele and the mutated allele with 116 bp deletion (Fig. 3D,E), indicating that this transgenic line could be a heterozygous *pds* mutant. Using our approach, most (28 out of 30, 93.3%) of the *pds* mutant plants were homozygotes, only 2 transgenic lines (6.7%) were considered as heterozygotes (Table 1). These results implies that the generation of DNA double stranded breaks (DSB) by Cas9 may occurred in an early stage in the regeneration T0 poplars from transgenic calli.

## Discussion

In a most recent report, the CRISPR/Cas9 system was delivered into sweet orange (*Citrus sinensis*) by transient infiltration of *A. rhizogenes*^25^, but stable transformation remains a preferred method for delivering nuclease constructs into wood species. To our knowledge, we first successfully applied a CRISPR/Cas9 approach for obtaining stable transgenic woody plants, and demonstrated that this system can efficiently and site-specifically mutate endogenous genes. Our data suggest that, by means of the CRISPR/Cas9 technology, locus-specific gene editing has become a routine practice not only in model plants and crops, but also in trees.

The multiple sites editing by the simultaneous expression of two or more sgRNAs has been reported in *Arabidopsis*^6^,^26^, rice^15^ and tomato^13^. But the pervious ways to delivery sgRNAs into plants are based on co-transformation of more than one plasmid, and relatively low efficiency, which restrict the application of multiple genes editing by CRISPR/Cas9 in plant genome. The pYLCRIPSR/Cas9 multiplex genome targeting vector system is designed to introduce to introduce multiple sgRNA expression cassettes by single vector transformation^23^. In this study, we used this vector system to successfully target multiple sites of the *PtoPDS* gene in poplar. Thus, one can foresee an application of the CRISPR/Cas9 system for knocking-out of whole gene family, as shown in rice^23^.

Besides, it is possible that the insertion or deletion mediated by single sgRNA guided Cas9 did not create a frameshift of target gene, consequently no defect in phenotype. Actually, the indels such as −3 bp or +1/−1  bp, which theoretically not lead to frameshift, were detected in the target 2 region (Fig. 3A). Because there are 4 sgRNAs for one locus being used in our experiment, the possibility that the changes in 3–4 target regions consequently escaped from frameshift is extremely low. Based on our data, we did not find that a transgenic plant with indels in the *PtoPDS* gene was still green. Therefore, the rate of modifications on multiple target sites affecting the reading frame was much lower in our results.

Our approach can also generate homozygous knockout mutation in predicted loci in T0 generation of transgenic poplar (Fig. 3), which is very important for genetic modification of trees with a long life span. With the advantages of both high efficiency in editing target gene sequence and synchronous mutation in two homologous chromosomes, poplar knockout mutants of interested genes could be generated in a very short time (approximately two months) and with a high ratio using the CRISPR/Cas9 method. Thus, our study proves that, using the CRISPR/Cas9 system, it is not only possible to investigate the function and mechanism of genes in woody trees, but also raises the possibility to construct a mutant library for poplar using the CRISPR/Cas9 system, instead of the T-DNA method used in *Arabidopsis*.

## Additional Information

**How to cite this article**: Fan, D. *et al*. Efficient CRISPR/Cas9-mediated Targeted Mutagenesis in Populus in the First Generation. *Sci. Rep*. **5**, 12217; doi: 10.1038/srep12217 (2015).

## Supplementary Material

Supplementary Information

## Figures and Tables

**Table 1:** Determination of mutation types in transgenic T0 poplar plants.

Target gene	No. of plants examined	No. of plants with mutations	Mutation rate (%)	Putative homozygous		Putative heterozygous	
				Number	%	Number	%
*PtoPDS*	59	30	51.7	28	93.2	2	6.7
CK	42	0	0	ND	ND	ND	ND

CK, Empty vector. ND, Not determined.
