# Supplementary material for: Efficient CRISPR/Cas9-mediated Targeted Mutagenesis in Populus in the First Generation
Source: Sci Rep. 2015 Jul 20;5:12217. doi: 10.1038/srep12217 (PMC4507398; doi:10.1038/srep12217)

Efficient CRISPR/Cas9-mediated Targeted Mutagenesis in Populus in the First Generation

Authors: Di Fan, Tingting Liu, Chaofeng Li, Bo Jiao, Shuang Li, Yishu Hou, Keming Luo

Supplementary information

Table S1. Statistical information describing the mutant alleles of *pds* in transgenic poplars.

	Plant ID	No. of clones sequenced	No. of clones with mutant alleles	No. of different mutant alleles
PtoPDS sgRNA1	1	18	0	0
	2	7	1	1
	3	10	10	4
	4	6	6	3
	5	6	6	2
	6	6	6	2
	7	6	6	3
	8	6	2	1
	Total	65	37	8
PtoPDS sgRNA2 &. sgRNA3	1	23	19	1
	2	13	13	6
	3	20	20	6
	4	12	8	3
	5	9	9	3
	6	12	12	5
	7	12	12	6
	8	11	7	3
	Total	112	100	17
PtoPDS sgRNA4	1	5	0	0
	2	6	0	0
	3	10	0	0
	4	6	0	0
	5	4	0	0
	6	6	0	0
	7	6	0	0
	8	5	0	0
	Total	48	0	0

Figure S1. Genomic sequence of the *PtoPDS* gene.

> *PtoPDS*

gttgaatttggttttggagaaATGAGTGCATTGAACTTGAGCTGGCATAGTAAATCATTA
GACTCTCAAGTTGCCTTGAGATGTGGCGCTTATCCTACTTGTTCTCACCAAA
CGAATGCACTAGCTTTTAGAGGCAGTGAATCAATGGGCCATTCTTTGAAATT
CCCATTTGGAAATTCTTCTGCTAAAACAAGACTAAGGAATCATATCCGCCCT
CCTTTGCGGGTgcgtagttctctacactacagggtaattattagttgccaatcaatacgtgaaaatttggggtgatctt
ttgtctacgctgtaggtTGTCTGTATGGACTATCCAAGACCGGACCTTGATAACACGG
TGAATTTCTTAGAGGCTGCCTTGTTATCTTCATCCTTTCGTTCTTCTCCGCGT
CCAGCTAAACCATTAAATGTTGTCATTGCTGGTGCAGgtgatgaaatcttatcctttttttgta
tgggaaaaaactgtgttgattatttagattgatttctatctatctgtaaaactttttcgttgaaatattgtcctcgtgtttaattaataat
tttgaagctcgtactttcacaagatttttgcatgtactcttgagagattgtgcgatacttactcgtgtgtgactaaaaattttccttat
tttttcagGTTTGGCGGGTTTATCGACTGCAAAATACTTGGCAGATGCGGGCCAT
AAGCCTATATTGCTTGAAGCAAGAGATGTTTTAGGTGGAAAGGTGGCTGCA
TGGAAAGATGATGATGGAGACTGGTACGAGACAGGCTTGCATATATTCTTT
GGGGCATATCCAAATGTGCAGAATCTTTTTGGTGAACTTGGTATCAATGATA
GGTTGCAATGGAAGGAGCATTCTATGATATTTGCAATGCCAAATAAGCCAG
GAGAATTCAGTCGATTTGATTTTCCTGAAGTTCTCCCTGCACCATTAAATG

Figure S2. Two copies of the *PtoPDS* gene were knocked-out in one independent transgenic line by the CRISPR/*Cas9* system. All of the sequences shown are from the transgenic line 3. The stars indicate the sequences of the second *PtoPDS* copy. The triangles indicate the nucleotide polymorphism sites between two copies of the *PtoPDS* genes.

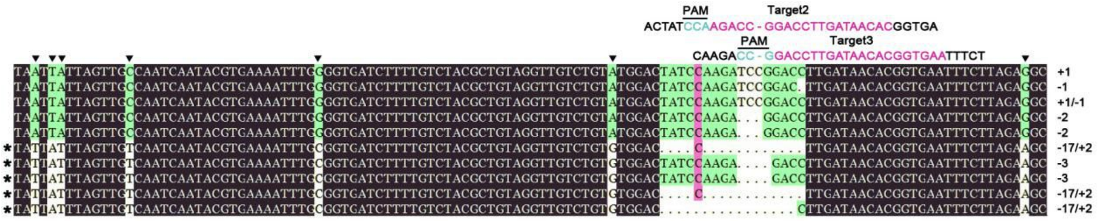

Supplement: Supplementary Information [file srep12217-s1.pdf]
